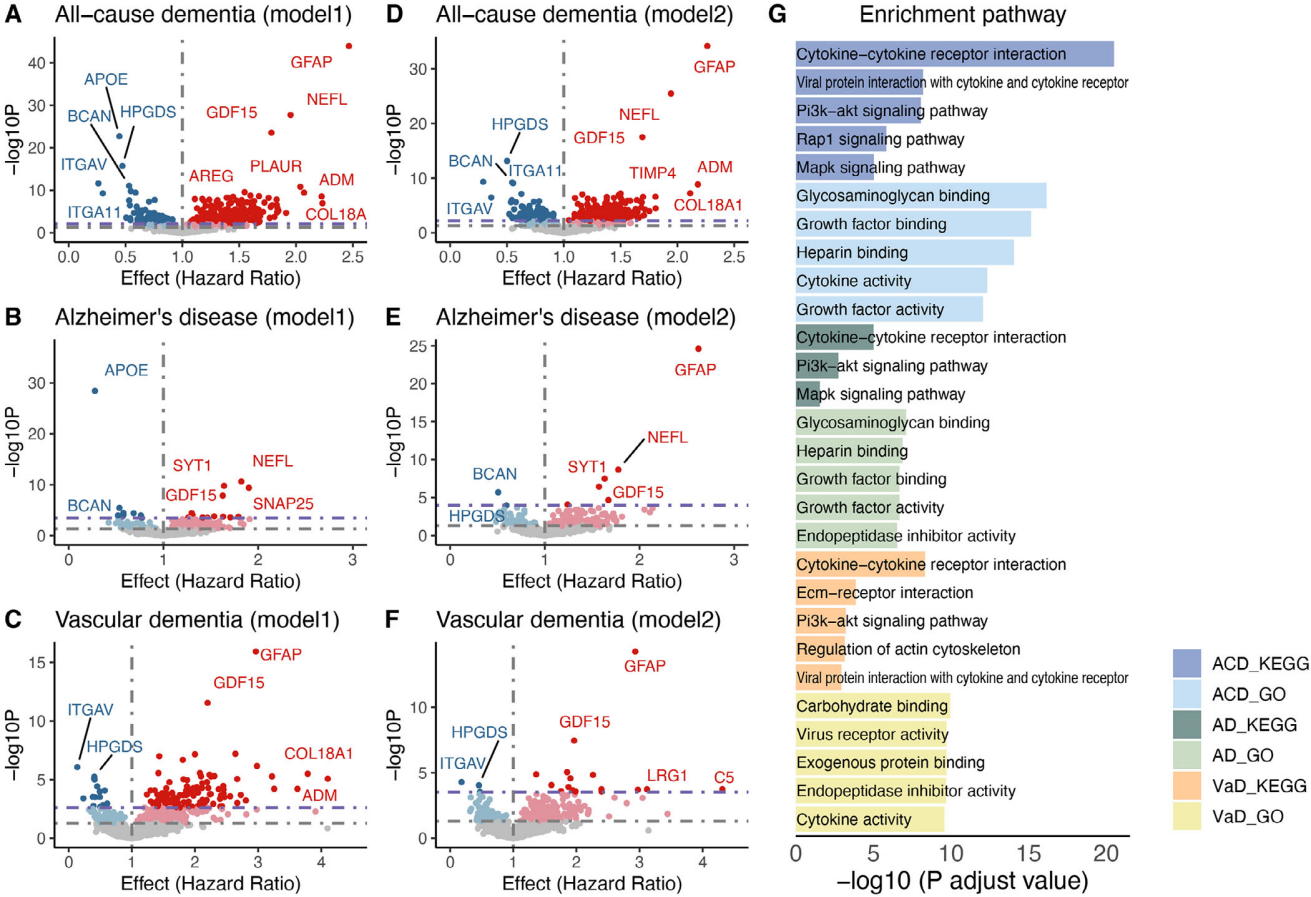# Plasma proteomics reveals novel protein signatures associated with dementia in chronic pain individuals: a prospective cohort study

**DOI:** 10.1002/alz70862_110859

**Published:** 2025-12-23

**Authors:** Pei‐Yang Gao, Ouyang Chen, Yi Tang, Xiaoduo Liu

**Affiliations:** ^1^ Xuanwu Hospital Capital Medical University, Beijing, Beijing China; ^2^ Department of Neurology & Innovation Center for Neurological Disorders, Xuanwu Hospital, Capital Medical University, National Center for Neurological Disorders, Beijing, Beijing China; ^3^ Xuanwu Hospital, Capital Medical University, Beijing, Beijing China

## Abstract

**Background:**

The relationship between chronic pain and Alzheimer’s disease (AD) remains poorly understood, despite growing evidence suggesting shared molecular pathways. Mass spectrometry‐based proteomics enables simultaneous analysis of thousands of proteins with high precision, providing advantages over traditional targeted methods. This study aimed to identify distinct plasma protein profiles that may link chronic pain to AD pathogenesis and potentially serve as early biomarkers.

**Method:**

We analyzed 2,920 plasma proteins from the UK Biobank. Chronic pain participants were identified through self‐reported pain lasting over three months at baseline. Cox proportional hazards models assessed longitudinal associations between proteins and risk of all‐cause dementia (ACD), AD, and vascular dementia (VaD) in chronic pain individuals. Kyoto Encyclopedia of Genes and Genomes (KEGG) and Gene Ontology (GO) enrichment analyses were performed to identify relevant biological pathways and processes.

**Results:**

A total of 20,932 chronic pain individuals (mean age: 57.51; 55.60% female), including 695 ACD, 332 AD, and 144 VaD cases, were followed for a median of 13.54 (SD 2.45) years. Among 2,920 analyzed proteins, after false discovery rate correction, GFAP showed the strongest association with all dementia types (ACD: HR=2.26; AD: HR=2.62; VD: HR=2.93). For ACD, NEFL (HR=1.94), GDF15 (HR=1.69) showed increased risk, while HPGDS (HR=0.50) and ITGAV (HR=0.29) showed protective effects. For AD, NEFL (HR=1.78), SYT1 (HR=1.63), and GDF15 (HR=1.57) were risk factors, while BCAN (HR=0.51) was protective. For VD, SPON2 (HR=2.26), GDF15 (HR=1.96), NEFL (HR=1.85), and FABP1 (HR=1.36) showed significant associations. Pathway analyses revealed shared enrichment in cytokine‐cytokine receptor interaction and Pi3k‐akt signaling across all dementia types. ACD showed specific enrichment in viral protein interaction and MAPK signaling pathways. AD was distinctly enriched in glycosaminoglycan and growth factor binding, while VD showed unique enrichment in ECM‐receptor interaction and regulation of actin cytoskeleton.

**Conclusion:**

Our study revealed distinct protein signatures linking chronic pain to dementia risk, with GFAP as a key marker across all dementia types. The enriched cytokine‐cytokine receptor interaction and Pi3k‐akt signaling pathways suggest chronic pain may accelerate neurodegeneration through neuroinflammation. These findings highlight potential mechanisms connecting chronic pain to cognitive decline and identify promising biomarkers for early dementia detection in chronic pain patients.